# Characterizing Race-Based Differences in Antisynthetase Syndrome Interstitial Lung Disease

**DOI:** 10.3390/biomedicines14030543

**Published:** 2026-02-27

**Authors:** Faye Pais, Caroline M. Cook, Stephen Simeone, Jessica A. Peterson, Kristina Akopyan, Maya Shamash, Christopher Harden, Bruno Hochhegger, Raju Reddy, Diana C. Gomez-Manjarres

**Affiliations:** 1 Division of Pulmonary, Critical Care and Sleep Medicine, Department of Medicine, University of Florida, 1600 SW Archer Rd, Gainesville, FL 32610, USAdiana.gomezmanjarres@medicine.ufl.edu (D.C.G.-M.); 2 Department of Pulmonary, Critical Care, and Sleep Medicine, Malcom Randall Veterans Administration Medical Center, 1601 SW Archer Rd, Gainesville, FL 32610, USA; 3 Ross School of Business, University of Michigan, 701 Tappan Ave, Ann Arbor, MI 48109, USA; 4 Department of Kinesiology, New Mexico State University, Las Cruces, NM 88003, USA; 5 North Florida Foundation of Research and Education, Malcom Randall Veterans Administration Medical Center, 1601 SW Archer Rd, Gainesville, FL 32610, USA; 6 Division of Hospital Medicine, Department of Medicine, University of Florida, 1600 SW Archer Rd, Gainesville, FL 32610, USA; 7 Department of Mechanical and Aerospace Engineering, University of Florida, 939 Center Drive, Gainesville, FL 32611, USA; 8 Department of Radiology, University of Florida, 1600 SW Archer Rd, Gainesville, FL 32611, USA; 9 Division of Pulmonary and Critical Care Medicine, Department of Medicine, University of Texas, Austin, TX 78712, USA

**Keywords:** antisynthetase syndrome, interstitial lung disease, Black patients, race, high-resolution computed tomography, traction bronchiectasis

## Abstract

**Background**: Interstitial lung disease (ILD) is the most serious manifestation of antisynthetase syndrome (ASyS). Limited research has explored racial differences in the clinical presentation and outcomes of ASyS-ILD. This study compared clinical manifestations, pulmonary function, and outcomes between Black and White patients with ASyS-ILD. **Methods**: A retrospective analysis was conducted using electronic health records from 2010 to 2020. Patients diagnosed with ILD and positive antisynthetase antibodies were included (N = 66; 34 Black and 32 White). Demographics, comorbidities, clinical features, pulmonary function tests, chest imaging, and clinical outcomes were compared between races. **Results**: Black patients were younger at diagnosis (49.1 ± 10.8 vs. 55.1 ± 12.9 years, *p* = 0.043) and had reduced pulmonary function (*p* < 0.01). Black patients also had a higher prevalence of traction bronchiectasis (96.6% vs. 73.1%, *p* = 0.012) and obstructive sleep apnea (*p* = 0.025). There were no differences in the frequency of hospitalizations, intensive care unit admissions, or deaths between groups. Myositis was common in both groups, and the distribution of antisynthetase antibodies did not differ by race (*p* = 0.333). **Conclusions**: Black patients are younger at diagnosis, have reduced lung function, and have increased traction bronchiectasis compared to White patients with ASyS-ILD. There is a higher prevalence of obstructive sleep apnea amongst Black patients without differences in body mass index. There was no difference in mortality and the need for lung transplantation. This study highlights several key differences between Black and White patients with ASyS-ILD. Longitudinal prospective studies are crucial to rigorously investigate race-based differences in morbidity, mortality, and long-term outcomes among patients with ASyS-ILD.

## 1. Introduction

Interstitial lung disease (ILD) is the leading cause of death in antisynthetase syndrome (ASyS), occurring in an estimated 70–100% of patients [1] ASyS, a subtype of idiopathic inflammatory myopathies (IIMs), is defined by the presence of a unique set of autoantibodies that target the aminoacyl-tRNA synthetases (ARSs), a group of enzymes responsible for catalyzing the first step of protein translation [2] While ILD is the most critical manifestation of ASyS, other clinical features of the syndrome include inflammatory myopathy (myositis), arthritis, Raynaud phenomenon, mechanic’s hands, and fever [3,4,5,6,7].

The presence and severity of systemic involvement in ASyS vary based on antibody profile and race. ILD occurs in up to 70% of patients with anti-Jo1 antibodies, compared to 98–100% of those with anti-EJ or anti-OJ antibodies [1] Anti-PL7 and anti-PL12 antibodies are associated with more severe lung involvement, whereas anti-Jo1 is linked to more severe myositis [8,9]. Lung involvement in the Black race is disproportionately more severe, independent of antibody type [9]. In the broader context of ILD, Black patients are younger at diagnosis, have more severe disease, higher mortality rates, and are more likely to be affected by healthcare disparities [10].

Taken together, the Black race is a common adverse factor in both ASyS and ILD at large. Because ILD is the most serious manifestation of ASyS, understanding race-based differences can guide targeted screening and individualized treatments. Therefore, our study objective was to explore differences in presentation, demographics, comorbidities, clinical course, and outcomes between Black patients and White patients with ASyS-ILD.

## 2. Materials and Methods

### 2.1. Study Design

We performed a retrospective review of electronic medical record (EMR) data from a university-affiliated academic medical center. The study protocol was approved by the institution’s ethical review board.

### 2.2. Study Participants and Eligibility Criteria

The study cohort was identified by querying the EMR for all patients who received care between 2010 and 2020 [11]. Patients were included if their medical records indicated a diagnosis of ILD using the ICD-10 code J84.9 along with a positive test for at least one ARS, including Jo-1, PL-7, PL-12, EJ, or OJ.

### 2.3. Study Variables

Following initial cohort identification, trained clinical research coordinators manually reviewed the medical records to extract required variables.

Exposure: The primary exposure was race, based on self-reported data in the EMR. Patients were categorized as non-Hispanic Black (hereafter Black) or non-Hispanic White (hereafter White), in line with established reporting guidelines [12].

Demographic and Clinical Variables: Demographic data included gender, age, body mass index (BMI), and smoking status. Comorbid conditions included obstructive sleep apnea (OSA), gastroesophageal reflux disease, hypertension, thromboembolic disease, type II diabetes, coronary artery disease, atrial fibrillation, liver cirrhosis, and end-stage renal disease.

ASyS Clinical Features and Serology: The presence of known ASyS systemic features (arthritis, Raynaud’s phenomenon, mechanic’s hands, fever) was recorded. Myositis was defined as elevated creatine kinase (CK) or aldolase levels. ARS antibody status was confirmed using results from commercially available lab panels (ARUP Laboratories, RDL, Salt Lake City, UT, USA).

Pulmonary Function Tests (PFTs): PFTs were performed according to ATS/ERS (American Thoracic Society/European Respiratory Society) standardization guidelines [13]. The primary variables extracted were the percentage of predicted forced vital capacity (FVC), forced expiratory volume in 1 s (FEV1), total lung capacity (TLC), and diffusing capacity of the lungs for carbon monoxide (DLCO).

Imaging: ILD diagnosis was based on compatible high-resolution computed tomography (HRCT) findings. A pulmonologist identified an ILD diagnosis, which was verified by a dedicated chest radiologist.

Course and Outcomes: The clinical course of ASyS-ILD was described based on the frequency of hospitalizations and intensive care unit (ICU) admissions. Long-term outcomes were defined as lung transplant or death.

### 2.4. Data Sources and Measurement

The EMR queries were formulated in consultation with the institution’s Integrated Data Repository (IDR) team, and data extraction was performed by the IDR consultant. ILD status was identified by qualified physicians and was documented in the EMR using ICD-10 code J84.9. ARS antibodies were tested using commercially available test panels (ARUP Laboratories, RDL).

Pulmonary Function Tests (PFTs): PFTs were performed according to ATS/ERS standardization guidelines [13]. The primary variables extracted were FVC, FEV1, TLC, and DLCO.

Imaging: ILD was evaluated using an HRCT of the chest. A pulmonologist confirmed the ILD diagnosis following EMR extraction, which was independently verified by a dedicated chest radiologist. HRCT scans were evaluated for radiographic pattern and severity based on ATS/ERS criteria [14]. ILD extent was scored using the Kazerooni system, which evaluates three lung zones (upper, middle, and lower) for a maximum possible score of 12 [15]. Two radiologists independently reviewed all imaging, with a third senior radiologist resolving any discordant interpretations to determine a final consensus severity score.

### 2.5. Bias and Study Size

This study was subject to limitations commonly found in retrospective and single-site study designs, including potential selection bias and misclassification bias. Selection bias may have occurred due to the inclusion of patients with documented ILD and available ARS antibody testing within the EMR. To mitigate this misclassification, ILD diagnoses were established by qualified physicians and identified using standardized ICD-10 coding. Laboratory data were obtained from commercially available ARS antibody panels, reducing variability in testing methodology. Information bias related to incomplete or missing data was possible. Hence, analyses were restricted to variables with sufficient documentation. The sample size was determined by the number of eligible patients meeting the inclusion criteria during the study period. A priori power calculation was not performed. ARS antibody titer data were not collected, and such titer-related correlations were not possible.

### 2.6. Quantitative Variables

Descriptive statistics were used to summarize data. Means and standard deviations (±SD) were calculated and reported for continuous variables, while counts and percentages were determined for categorical variables.

### 2.7. Statistical Methods

Comparisons between Black and White patient groups were performed using either a chi-square (X^2^) test for categorical data with 3 or more categories, Fisher’s exact test for dichotomous categorical variables, or independent samples *t*-tests when examining continuous variables. All analyses were conducted using SPSS version 29.0, with statistical significance set at *p* < 0.05. Missing data patterns were assessed and categorized as missing completely at random, missing at random, or missing not at random. The extent and pattern of missingness were reported for all key variables stratified by race. For variables with <10% missing data, complete case analysis was performed. For variables with 10–50% missingness, multiple imputations using chained equations were employed to generate five imputed datasets with results pooled using Rubin’s rules. Variables with >50% missing data were excluded.

### 2.8. Data Management and Cleaning Methods

Sensitive information was secured on the institution’s encrypted electronic data storage platform. Only de-identified data was downloaded for analysis. Data cleaning was performed by manually verifying ARS antibody profiles, race, and ILD status.

### 2.9. Data Linkage

Probabilistic linkage to external datasets was not performed.

## 3. Results

### 3.1. Demographics and Comorbidities

We identified 66 patients with interstitial lung disease (ILD) and a positive antisynthetase (ARS) antibody. The cohort consisted of 34 Black (51.5%) and 32 White (48.5%) patients. Black patients were significantly younger than White patients. While a trend toward a higher body mass index (BMI) was observed in Black patients, the difference did not reach statistical significance (*p* = 0.088). The majority of patients were never smokers (60.6%). Obstructive sleep apnea was significantly more prevalent among Black patients than among White patients (*p* = 0.025). Hypertension was the most common comorbidity in both groups (Table 1).

### 3.2. Clinical Characteristics and Antibody Profiles

Antisynthetase syndrome clinical characteristics did not differ significantly between Black and White patients. After ILD, myositis was the most common manifestation (45/66, 66.7%), followed by arthritis (25/66, 37.9%), Raynaud phenomenon (14/66, 21.2%), and mechanic’s hands (12/66, 18.2%). Regarding ARS antibodies, anti-Jo-1 was the most prevalent (44/66, 66.7%), followed by anti-PL-12 (14/66, 21.2%), anti-PL-7 (5/66, 7.6%), and anti-EJ (3/66, 4.5%). Anti-OJ antibody was not detected. The distribution of antibody positivity did not significantly differ between Black and White patients, *p* = 0.333 (Table 2).

### 3.3. Baseline Pulmonary Function Tests

Baseline PFTs were available for 56 of 66 patients (Table 3). Black patients had significantly lower baseline lung function compared to White patients, with reduced mean FVC % (55.0% vs. 66.5%; *p* = 0.008), FEV1% (57.5% vs. 69.4%; *p* = 0.009), and TLC % (54.1% vs. 66.0%; *p* = 0.006). Although DLCO% was also lower in Black patients, this difference did not reach statistical significance (*p* = 0.063).

### 3.4. Baseline Imaging Characteristics

Baseline HRCT scans were available for 56 of the 66 patients (26 White and 30 Black). Inflammatory imaging patterns predominated in both groups. All patients had GGOs, mainly in the periphery and lower zones. GGOs with consolidation were seen in nine patients. Reticular opacities were present in 100% of Black patients and 88.4% of White patients. Traction bronchiectasis was significantly more common in Black patients than in White patients (96.6% vs. 73.1%; *p* = 0.012; Table 4).

### 3.5. Disease Course and Outcomes

Among the 66 patients, 34 (51.5%) were hospitalized at least once (Table 5). Black patients had higher rates of multiple hospitalizations (≥3 admissions: 26.5% vs. 21.9%) and intensive care unit admissions (23.5% vs. 15.6%) compared to White patients. The overall mortality rate was 25.8% (17/66), with similar rates in Black (26.5%) and White (25.0%) patients. Known causes of death included ILD exacerbation (n = 1), infection (n = 2), and pulmonary hypertension with right ventricular failure (n = 3). Three patients underwent lung transplantation (one Black and two White).

## 4. Discussion

This study identifies important race-based differences between Black and White patients with ASyS-ILD. Black patients were significantly younger at diagnosis than White patients, a finding consistent with the broader ILD literature [10,16,17]. Black patients demonstrated significantly worse baseline pulmonary function, with lower FVC, FEV1, and TLC percentages compared to White patients. Lung function differences did not vary with ARS antibody type. Our findings align with previous reports of more severe lung disease in Black patients with ASyS-ILD occurring independent of ARS antibody profile [9]. Similarly, ASyS-ILD in Afro-Caribbean patients had lower lung function and higher dyspnea scores compared to Caucasians [18]. Although our study did not investigate genetic differences, these findings suggest that genetic differences exist between races regarding pulmonary fibrosis development, which can be explored further with subsequent studies. There is increasing evidence that genetic factors can contribute to both disease onset and progression in pulmonary fibrosis [19].

Importantly, while all Black patients had reticular opacities (vs. 88.4% in the White group), traction bronchiectasis was more prevalent among the Black group. Traction bronchiectasis represents more advanced scarring and is linked to increased all-cause mortality in patients with connective tissue-associated ILDs [20]. Recurrent respiratory infections, smoking status, and duration of immunosuppression can impact imaging findings. Most patients in our study were non-smokers, with no statistically significant difference in hospitalizations, although data regarding immunosuppression and respiratory infections were not collected. In Black patients, lower lung function and increased fibrosis at baseline could suggest more advanced ILD at diagnosis, either due to diagnostic delays or predisposition for more fibrotic phenotypes. Baseline younger age at diagnosis could also indicate earlier disease onset and overall increased total disease duration.

Black patients with ILD have a shorter time to first hospitalization and death. While these associations could not be examined in our cohort, larger dedicated studies in ASyS-ILD are critically important. Although the differences in death between White and Black patients with ASyS-ILD did not reach statistical significance, there were prominent numerical differences in infections, ILD exacerbations, and PH/RV failure between the groups. Black patients may be at higher risk of these significant causes of death, which can also be explored further with studies of a large cohort size. Extrapolating from the other autoimmune ILD literature, Black patients with systemic sclerosis (SSc)-related ILD have pulmonary involvement at a younger age and have a higher incidence of emergency department visits when compared to patients of other racial backgrounds [21,22]. African ancestry was also found to be a risk factor for developing SSc and idiopathic inflammatory myositis [23]. Other investigations have demonstrated that sarcoidosis is not only more prevalent in patients of African ancestry but that these patients also experience more severe pulmonary parenchymal involvement, more advanced radiographic imaging, and more frequent hospitalizations [24]. Taken together, there is substantial evidence to suggest race is an important factor in a broad range of ILDs associated with systemic autoimmune rheumatologic diseases. Our paper provides support to highlight the importance of race in ASyS-ILD.

OSA was significantly higher in the Black patient population. Several investigations have examined the prevalence of OSA in patients with both idiopathic pulmonary fibrosis (IPF) and other fibrotic forms of ILD and have found this population to be burdened with higher rates of OSA [25]. Lee and colleagues present that higher BMIs, advanced age, and comorbid diabetes mellitus were all independent risk factors for OSA in patients with various types of ILD [26,27]. Interestingly, there was no significant difference in BMI or smoking between our Black and White patients, including a non-significant trend towards significantly higher BMI in the Black patient cohort. Increased apnea–hypopnea index is negatively prognostic in ILD patients, suggesting that OSA may potentiate more deleterious outcomes regardless of ILD subtype [28]. Furthermore, the duration of immunosuppressive therapy in fibrotic ILD has been linked to alterations in FEV1 [29], while in non-IPF ILD, a reduced TLC is independently predictive of OSA risk, and the observed nocturnal hypoxemia is reversible with non-invasive positive pressure ventilation [30]. In our cohort of non-IPF, ASyS-ILD patients, Black patients had lower FEV1 and TLC at baseline and were on immunosuppressive therapy. Additional contributors could also include altered respiratory mechanics and lifestyle factors [31,32] While no direct causal links are possible, our study supports the need for future investigations in this area.

Race-based socioeconomic disparities are especially burdensome in Black ILD patients. Disadvantaged communities and their associated healthcare infrastructure have limited access to radiology centers, spirometry equipment, comprehensive health insurance, and subspecialty medical providers, delaying timely diagnosis and treatment [10]. ILD by itself is prone to diagnostic delays [33], and socioeconomic discrepancies become added challenges.

Our study highlights several key differences between Black and White patients with ASyS-ILD. However, lack of longitudinal follow-up, repeat testing data, reliance on EMR documentation, and patient reports limit generalizability and causal conclusions. Smaller size from potential underreporting is another challenge. These factors in combination may have reduced our ability to detect other significant differences between the two groups.

## 5. Conclusions

Black patients with ASyS-ILD were younger at diagnosis, had lower baseline lung function, and had more severe HRCT evidence of fibrosis. While all Black patients had reticular opacities, traction bronchiectasis was significantly more prevalent in the Black subgroup. OSA was another finding that occurred more commonly in Black patients, irrespective of BMI status. Our study recognizes the presence of race-based differences in ASyS-ILD patients. Dedicated prospective, multicenter longitudinal studies are required to critically understand and validate these findings.

## Figures and Tables

**Table 1:** Demographics and comorbidities of black and white patients with antisynthetase syndrome.

Variable	Total (N = 66)	White (N = 32)	Black (N = 34)	*p*-Value
**Demographics**				
Female (%)	45 (68.2%)	22 (68.7%)	23 (67.6%)	0.923
Age (years)	52.0 ± 12.2	55.1 ± 12.9	49.1 ± 10.8	**0.043**
BMI (kg/m^2^)	29.8 ± 6.46	28.4 ± 5.8	31.1 ± 6.9	0.088
Smoking—Current	5 (7.6%)	5 (15.6%)	0 (0.0%)	0.056
Smoking—Former	21 (31.8%)	9 (28.8%)	12 (37.5%)	
Smoking—Never	40 (60.6%)	18 (56.3%)	22 (64.7%)	
**Comorbidities**				
Obstructive Sleep Apnea	6 (9.1%)	0 (0.0%)	6 (17.6%)	**0.025**
Thromboembolic Disease	10 (15.2%)	6 (18.8%)	4 (11.8%)	0.327
GERD	23 (34.8%)	11 (34.4%)	12 (35.3%)	>0.999
Hypertension	39 (59.1%)	17 (53.1%)	22 (64.7%)	0.339
Type II Diabetes	16 (24.2%)	5 (15.6%)	11 (32.4%)	0.113
Coronary Artery Disease	5 (7.6%)	1 (3.1%)	4 (11.8%)	0.185
Atrial Fibrillation	9 (13.6%)	6 (18.8%)	3 (8.8%)	0.24
Cirrhosis	1 (1.5%)	1 (3.1%)	0 (0.0%)	0.299
End Stage Renal Disease	4 (6.1%)	3 (9.3%)	1 (2.9%)	0.274

Continuous variables are specified using means and standard deviations. Categorical variables are specified as counts and percentages. Note: BMI—body mass index, GERD—gastroesophageal reflux disease. Bold values indicate significant difference where *p* < 0.05.

**Table 2:** Antisynthetase syndrome characteristics of Black and White patients.

Category	Variable	Total (N = 66)	White (N = 32)	Black (N = 34)	*p*-Value
Clinical Features	Raynaud’s Phenomenon	14 (21.2%)	7 (21.9%)	7 (20.5%)	0.898
	Mechanic’s Hands	12 (18.2%)	7 (21.9%)	5 (14.7%)	0.450
	Arthritis	25 (37.9%)	15 (46.9%)	10 (29.4%)	0.144
Elevated Muscle Enzymes		45 (68.2%)	19 (59.4%)	26 (76.5%)	0.136
Autoimmune Serology	anti-Jo-1	44 (66.7%)	24 (75.0%)	20 (58.8%)	0.333
	anti-PL-12	14 (21.2%)	4 (12.5%)	10 (29.4%)	
	anti-PL-7	5 (7.6%)	3 (9.4%)	2 (5.9%)	
	anti-EJ	3 (4.5%)	1 (3.1%)	2 (5.9%)	
	anti-OJ	0 (0.0%)	0 (0.0%)	0 (0.0%)	

Note: Categorical variables are specified as percentages; Jo-1 = anti-histidyl tRNA synthetase; PL-12 = anti-alanyl tRNA synthetase; PL-7 = anti-threonyl tRNA synthetase; EJ = anti-glycyl tRNA synthetase; OJ = anti-isoleucyl tRNA synthetase.

**Table 3:** Pulmonary Function Test characteristics of Black and White patients with antisynthetase syndrome.

Variable	Total (N = 56)	White (N = 25)	Black (N = 31)	*p*-Value
FVC (%)	60.1 ± 17.5	66.5 ± 18.0	55.0 ± 13.6	**0.008**
FEV1 (%)	62.8 ± 17.5	69.4 ± 17.1	57.6 ± 16.1	**0.009**
TLC (%)	59.2 ± 24.2	66.0 ± 18.1	54.1 ± 12.1	**0.006**
DLCO Hg (%)	50.7 ± 24.2	56.6 ± 24.6	44.6 ± 22.8	0.063

Note: Continuous variables are specified using means with standard deviations; FVC = forced vital capacity; FEV1 = forced expiratory volume in 1 sec; TLC = total lung capacity; DLCO Hg = diffusion capacity of lung for carbon monoxide corrected for hemoglobin; bolded values indicate significant difference where *p* < 0.05.

**Table 4:** High resolution computed tomography characteristics of Black and White Patients with antisynthetase syndrome.

Finding	Total (N = 56)	White (N = 26)	Black (N = 30)	*p*-Value
Traction Bronchiectasis	48 (85.7%)	19 (73.1%)	29 (96.6%)	**0.012**
Consolidation	9 (26.8%)	5 (19.2%)	4 (33.3%)	0.549
GGO	55 (98.2%)	25 (96.2%)	30 (100.0%)	0.278
Distribution of GGO—Upper	0 (0.0%)	0 (0.0%)	0 (0.0%)	0.839
Distribution of GGO—Lower	49 (87.5%)	23 (88.4%)	26 (86.6%)	
Distribution of GGO—Diffuse	7 (12.5%)	3 (11.5%)	4 (13.3%)	
Distribution of GGO—Perihilar	0 (0.0%)	0 (0.0%)	0 (0.0%)	
Distribution of GGO—Peripheral	55 (98.2%)	26 (100%)	29 (96.6%)	0.348
Distribution of GGO—Diffuse	1 (1.8%)	0 (0.0%)	1 (3.3%)	
Reticular Changes	53 (94.6%)	23 (88.4%)	30 (100.0%)	0.056
Honeycombing	7 (12.5%)	4 (15.4%)	3 (10.0%)	0.543
HRCT Severity Index—<5%	7 (12.5%)	6 (18.8%)	1 (3.3%)	0.204
HRCT Severity Index—5–20%	14 (25.0%)	7 (21.8%)	7 (26.9%)	
HRCT Severity Index—21–40%	17 (30.4%)	6 (18.8%)	11 (32.4%)	
HRCT Severity Index—41–60%	14 (25.0%)	5 (15.6%)	9 (26.5%)	
HRCT Severity Index—61–80%	4 (7.1%)	2 (7.6%)	2 (5.9%)	
HRCT Severity Index—81–100%	0 (0.0%)	0 (0.0%)	0 (0.0%)	

Categorical variables are specified as percentages; HRCT = high resolution computed tomography; GGO = ground-glass opacity; bolded values indicate significant difference where *p* < 0.05.

**Table 5:** Clinical course and outcomes of Black and White patients with antisynthetase syndrome.

Variable	Total (N = 66)	White (N = 32)	Black (N = 34)	*p*-Value
**Clinical Course**				
*Hospitalizations—0	29 (43.9%)	15 (46.9%)	14 (41.2%)	0.94
*Hospitalizations—1	13 (19.7%)	6 (18.8%)	7 (20.6%)	
*Hospitalizations—2	5 (7.6%)	2 (6.3%)	3 (8.8%)	
*Hospitalizations—3 or more	16 (24.2%)	7 (21.9%)	9 (26.5%)	
*Hospitalizations—Unknown	3 (4.5%)	2 (6.3%)	1 (2.9%)	
**ICU Admissions—Yes	13 (19.7%)	5 (15.6%)	8 (23.5%)	0.38
**ICU Admissions—No	42 (63.6%)	22 (68.8%)	20 (58.8%)	
**ICU Admissions—Unknown	11 (16.7%)	5 (15.6%)	6 (17.6%)	
**Outcomes**				
Transplant	3 (4.5%)	2 (6.3%)	1 (2.9%)	0.124
Cause of Death—Infection	2 (11.8%)	0 (0.0%)	2 (22.2%)	0.23
Cause of Death—ILD Exacerbation	1 (5.9%)	1 (12.5%)	0 (0.0%)	
Cause of Death—PH/RV Failure	3 (4.5%)	1 (12.5%)	2 (22.2%)	
Cause of Death—Other	11 (64.7%)	6 (75.0%)	5 (55.6%)	
Death	17 (25.8%)	8 (25.0%)	9 (26.5%)	0.891

Note: Categorical variables are specified as percentages; ICU = intensive care unit; ILD = interstitial lung disease; PH = pulmonary hypertension; RV = right ventricular; * 3 missing cases; ** 11 missing cases.

## Data Availability

The data presented in this study are available on request from the corresponding author.
